# Complete Genome Assembly of Reference Strain *Ochrobactrum anthropi* ATCC 49687

**DOI:** 10.1128/genomeA.00962-14

**Published:** 2014-09-25

**Authors:** T. D. Minogue, H. A. Daligault, K. W. Davenport, K. A. Bishop-Lilly, D. C. Bruce, P. S. Chain, O. Chertkov, S. R. Coyne, T. Freitas, K. G. Frey, J. Jaissle, G. I. Koroleva, J. T. Ladner, G. F. Palacios, C. L. Redden, Y. Xu, S. L. Johnson

**Affiliations:** a Diagnostic Systems Division (DSD), United States Army Medical Research Institute of Infectious Diseases (USAMRIID), Fort Detrick, Maryland, USA; b Los Alamos National Laboratory, Los Alamos, New Mexico, USA; c Naval Medical Research Center (NMRC)-Frederick, Fort Detrick, Maryland, USA; d Henry M. Jackson Foundation, Bethesda, Maryland, USA; e Center for Genome Sciences (CGS), USAMRIID, Fort Detrick, Maryland, USA

## Abstract

*Ochrobactrum anthropi* is an occasional cause of nosocomial infections; however, interest in the organism lies in its phylogenetic proximity to the genus *Brucella*. Here, we present the 4.9-Mb finished genome of *Ochrobactrum anthropi* ATCC 49687, most commonly used as an exclusionary reference organism.

## GENOME ANNOUNCEMENT

*Ochrobactrum anthropi* ATCC 49687 causes sporadic nosicomal human infection but is commonly found in environmental samples (1). The ATCC 49687 strain is most commonly used as a reference organism in phenotypic kits. Important in its own right, *O. anthropic* is largely used in phylogenomics and diagnostics as it is the closest relative to *Brucella* based on both DNA and protein analyses (2–4).

High-quality genomic DNA was extracted from a purified isolate using a QIAgen Genome Tip-500 at USAMRIID-DSD by growing a 100-ml culture to stationary phase and extracting nucleic acid as per manufacturer’s recommendations. Sequence data for the draft genome includes a combination of Illumina and 454 technologies (5, 6). For this genome assembly, we constructed and sequenced an Illumina library of 100-bp reads to high coverage (301-fold genome-coverage) and a separate long-insert paired-end library (average insert size, 7,704 ± 1,926-bp, run on Roche 454 Titanium platform to 30-fold genome-coverage). The two datasets were assembled together in Newbler (Roche) and the consensus sequences computationally shredded into 2-kbp overlapping fake reads (shreds). The raw reads were also assembled in Velvet and those consensus sequences computationally shredded into 1.5-kbp overlapping shreds (7). Draft data from all platforms was then assembled together with Allpaths and the consensus sequences computationally shredded into 10-kbp overlapping shreds (8). We then integrated the Newbler consensus shreds, Velvet consensus shreds, Allpaths consensus shreds, and a subset of the long-insert read-pairs using parallel Phrap (High Performance Software, LLC). Possible misassemblies were corrected and some gap closure accomplished with manual editing in Consed (9–11).

Automatic annotation for the *O. anthropi* ATCC 49687 genome utilized an Ergatis based workflow at Los Alamos National Laboratory (LANL) with minor manual curation. Annotation located 4,630 coding genes, 57 tRNAs, and 12 rRNAs. The final 4,901,165-bp assembly has 56.1% G+C content. To our knowledge, there is only one other complete genome of *O. anthropi* publicly available (strain ATCC 49118^T^); however, further investigation is warranted to understand the genetic relationship between the two strains (12).

### Nucleotide sequence accession numbers.

The final sequence has been deposited to GenBank under four accession numbers: CP008820 (Chr I), CP008819 (Chr II), CP008817 (pOAB1), and CP008818 (pOAB2).

## References

[1] Duran R Vatansever Ü Acunaş B Başaran ÜN 2009 *Ochrobactrum anthropi* bacteremia in a preterm infant with meconium peritonitis. Int. J. Infect. Dis. 13:e61–e63. 10.1016/j.ijid.2008.06.027 18842433

[2] Kern WV Oethinger M Marre R Kaufhold A Rozdzinski E 1993 *Ochrobactrum anthropi* bacteremia: report of four cases and short review. Infection 21:306–310. 10.1007/BF01712451 8300247

[3] Kulkarni G Dhotre D Dharne M Shetty S Chowdhury S Misra V Misra S Patole M Shouche Y 2013 Draft genome of *Ochrobactrum intermedium* strain M86 isolated from non-ulcer dyspeptic individual from India. Gut Pathog. 5:7. 10.1186/1757-4749-5-7 23557353 PMC3640943

[4] Velasco J Romero C López-Goñi I Leiva J Díaz R Moriyón I 1998 Evaluation of the relatedness of *Brucella* spp. and *Ochrobactrum anthropi* and description of *Ochrobactrum intermedium* sp. nov., a new species with a closer relationship to *Brucella* spp. Int. J. Syst. Bacteriol. 48:759–768. 10.1099/00207713-48-3-759 9734029

[5] Margulies M Egholm M Altman WE Attiya S Bader JS Bemben LA Berka J Braverman MS Chen Y-J Chen Z Dewell SB Du L Fierro JM Gomes XV Godwin BC He W Helgesen S Ho CH Irzyk GP Jando SC Alenquer MLI Jarvie TP Jirage KB Kim J-B Knight JR Lanza JR Leamon JH Lefkowitz SM Lei M Li J Lohman KL Lu H Makhijani VB McDade KE McKenna MP Myers EW Nickerson E Nobile JR Plant R Puc BP Ronan MT Roth GT Sarkis GJ Simons JF Simpson JW Srinivasan M Tartaro KR Tomasz A Vogt KA Volkmer GA Wang SH Wang Y Weiner MP Yu P Begley RF Rothberg JM 2005 Genome sequencing in microfabricated high-density picolitre reactors. Nature 437:376–380. 10.1038/nature03959 16056220 PMC1464427

[6] Bennett S 2004 Solexa Ltd. Pharmacogenomics 5:433–438. 10.1517/14622416.5.4.433 15165179

[7] Zerbino DR Birney E 2008 Velvet: algorithms for *de novo* short read assembly using de Bruijn graphs. Genome Res. 18:821–829. 10.1101/gr.074492.107 18349386 PMC2336801

[8] Butler J MacCallum I Kleber M Shlyakhter IA Belmonte MK Lander ES Nusbaum C Jaffe DB 2008 ALLPATHS: *de novo* assembly of whole-genome shotgun microreads. Genome Res. 18:810–820. 10.1101/gr.7337908 18340039 PMC2336810

[9] Ewing B Hillier L Wendl MC Green P 1998 Base-calling of automated sequencer traces using Phred. I. Accuracy assessment. Genome Res. 8:175–185. 10.1101/gr.8.3.175 9521921

[10] Ewing B Green P 1998 Base-calling of automated sequencer traces using Phred. II. Error probabilities. Genome Res. 8:186–194 9521922

[11] Gordon D Abajian C Green P 1998 Consed: a graphical tool for sequence finishing. Genome Res. 8:195–202. 10.1101/gr.8.3.195 9521923

[12] Chain PS Lang DM Comerci DJ Malfatti SA Vergez LM Shin M Ugalde RA Garcia E Tolmasky ME 2011 Genome of *Ochrobactrum anthropi* ATCC 49188^T^, a versatile opportunistic pathogen and symbiont of several eukaryotic hosts. J. Bacteriol. 193:4274–4275. 10.1128/JB.05335-11 21685287 PMC3147672

